# Circadian rhythmicity in patients with out-of-hospital cardiac arrest and ST-segment elevation myocardial infarction: a scoping review

**DOI:** 10.62838/jccm-2026-0029

**Published:** 2026-10-30

**Authors:** Hana Locihová, Petr Matouch, Jan Romba, Miroslava Kachlová

**Affiliations:** Department of Anaesthesiology, Resuscitation and Intensive Care Medicine, Faculty of Medicine, University of Ostrava,Czech Republic; Department of Nursing and Midwifery, Faculty of Medicine, University of Ostrava,Czech Republic; Department of Anaesthesiology, Resuscitation and Intensive Care Medicine, University Hospital Ostrava,Czech Republic

**Keywords:** Circadian rhythm, Out-of-hospital cardiac arrest, ST-segment elevation myocardial infarction

## Abstract

**Introduction:**

Out-of-hospital cardiac arrest (OHCA) and ST-segment elevation myocardial infarction (STEMI) are time-critical events that exhibit circadian patterns, which influence their incidence and clinical outcomes.

**Objective:**

To determine whether circadian rhythmicity influences the time of onset of OHCA and STEMI, as well as related clinical outcomes, in patients requiring prehospital emergency care and subsequent hospitalization.

**Design:**

A scoping review.

**Methods:**

The search process included four online databases: MEDLINE Complete, CINAHL Plus with Full Text, ScienceDirect, and ProQuest. The review included original articles published between January 2010 and December 2024.

**Results:**

Eighteen studies met the inclusion criteria. These revealed that the incidence of OHCA and STEMI exhibits significant circadian variability, with the highest incidence in the morning. OHCA often has a secondary afternoon/evening peak. The nighttime hours are associated with the lowest incidence. Patients with nighttime OHCA or STEMI have a lower probability of return of spontaneous circulation and worse one-month and one-year survival and neurological outcomes. This less favorable prognosis is not due to the time of day when the event occurs but rather due to limited bystander availability, lower rates of bystander cardiopulmonary resuscitation and defibrillation, and longer times to hospital intervention. Additionally, these events were more frequently recorded in winter, while weekly variations were insignificant.

**Conclusion:**

Circadian and seasonal factors influence the occurrence and clinical course of OHCA and STEMI, with worse outcomes for nighttime cases being more a consequence of limited availability than the time of the event itself.

## Introduction

Circadian rhythms are endogenous biological processes with a period of approximately 24 hours that regulate a wide range of physiological functions, including the sleep-wake cycle, metabolism, and cardiovascular health [1,2]. These rhythms modulate key cardiovascular parameters such as blood pressure, heart rate, and endothelial function, and their disruption increases the risk of acute events – myocardial infarction, stroke, arrhythmias, and sudden cardiac arrest, especially in the morning hours when there is an increase in sympathetic activity and hormonal oscillations [3,4].

ST-segment elevation myocardial infarction (STEMI) affects more than three million people worldwide each year, with approximately 72.7 cases per 100,000 population in Europe [5]. The incidence of out-of-hospital cardiac arrest (OHCA) in Europe is estimated at 67 to 170 cases per 100,000 [6].

OHCA and STEMI are acute, life-threatening conditions that are epidemiologically distinct, but share overlapping pathophysiological mechanisms. OHCA is characterized by an abrupt cessation of cardiac mechanical activity outside of a hospital setting. Survival and neurological outcomes depend on early OHCA recognition, initiation of bystander resuscitation, defibrillation, and rapid access to expert care [7,8]. STEMI is an acute transmural occlusion of a coronary artery that requires immediate reperfusion, as time to intervention directly affects survival and the risk of cardiac arrest [9]. Both conditions are therefore highly time-sensitive and necessitate optimized prehospital and hospital care.

Epidemiological studies show that the incidence rates of OHCA and STEMI exhibit significant circadian and seasonal patterns, which also impact patient clinical outcomes [10]. This overlap in temporal distribution may be partly explained by the fact that OHCA is predominantly of cardiac origin, most commonly related to underlying coronary artery disease and acute coronary syndromes, including STEMI [11]. However, study results vary by region [12]. The time of symptom onset has a direct effect on the organization and availability of prehospital emergency care. It has been documented that, compared to afternoon cases, it takes longer for patients with nighttime STEMI to arrive at the hospital and start reperfusion therapy [13]. Large-scale data analyses also confirm that emergency calls are not evenly distributed throughout the day and that and most categories, including cardiac events, show typical peaks in incidence[14].

The present review aimed to determine whether circadian rhythmicity affects the time of onset of OHCA and STEMI and to evaluate the association between the time of onset and clinical outcomes in patients requiring prehospital emergency care and subsequent hospitalization.

## Materials and methods

### Design

This scoping review was undertaken in accordance with the five stages of [15] scoping review framework: (1) identifying the research question; (2) identifying relevant studies; (3) study selection; (4) charting the data; and (5) collating, summarizing, and reporting the results. The Preferred Reporting Items for Systematic reviews and Meta-Analyses extension for Scoping Reviews (PRISMA-ScR) checklist guided reporting of this scoping review [16].

### Research question

How does circadian rhythmicity affect the time of onset of OHCA and STEMI and the related clinical outcomes of patients requiring prehospital emergency care and subsequent hospitalization?

### Inclusion and exclusion criteria

The scoping review included literature meeting the following criteria: (a) publication date between January 2010 and December 2024; (b) original articles published in peer-reviewed journals; (c) studies available in full text; (d) circadian rhythmicity in relation to OHCA and STEMI as the primary topic; and (e) English language. The exclusion criteria were as follows: (a) patient age less than 18 years; (b) review articles, case reports, or meta-analyses; and (c) studies focusing on other conditions in relation to circadian rhythmicity.

### Sources and search strategy

A systematic literature search was performed in MED-LINE Complete, CINAHL Plus with Full Text, ScienceDirect, and ProQuest databases. To define the study objective, the following keywords were used in various combinations, linked by Boolean operators: “prehospital care”, “emergency medical services”, “circadian rhythm”, “diurnal variation”, “out-of-hospital cardiac arrest”, “myocardial infarction”, “time of occurrence”, “mortality”, “survival rate”, and “hospital outcomes”. These primary keywords were linked by the Boolean operators AND/OR. The same search criteria were used for each database. The results of available relevant original papers were used to verify the predefined objectives. The above inclusion/exclusion criteria and search strategies were employed. Suitable literature identified was further analyzed.

### Study selection and data extraction

A total of 319 records were found across the selected databases. The search was conducted by three researchers and a librarian. The literature was evaluated independently by two researchers. Ninety-four articles were excluded due to duplication. In the first assessment, which was based on the titles and abstracts, 225 articles were examined. Of those, 174 were excluded because they were not relevant to the topic under study. In the second assessment, when the full texts were evaluated, another 33 studies were excluded from the remaining 51 articles because they did not meet the inclusion criteria. For the final 18 articles, reference lists were also reviewed to identify any additional studies. The articles were identified and classified in accordance with the PRISMA flow diagram (Figure 1). Primary extraction of data from the 18 selected studies included information on authors, country of origin, research design, study objectives, forms of data processing, evaluated parameters, research results, and conclusions (Tables 1, 2). This was followed by an analysis of the monitored variables and synthesis of the conclusions.

**Fig. 1.**
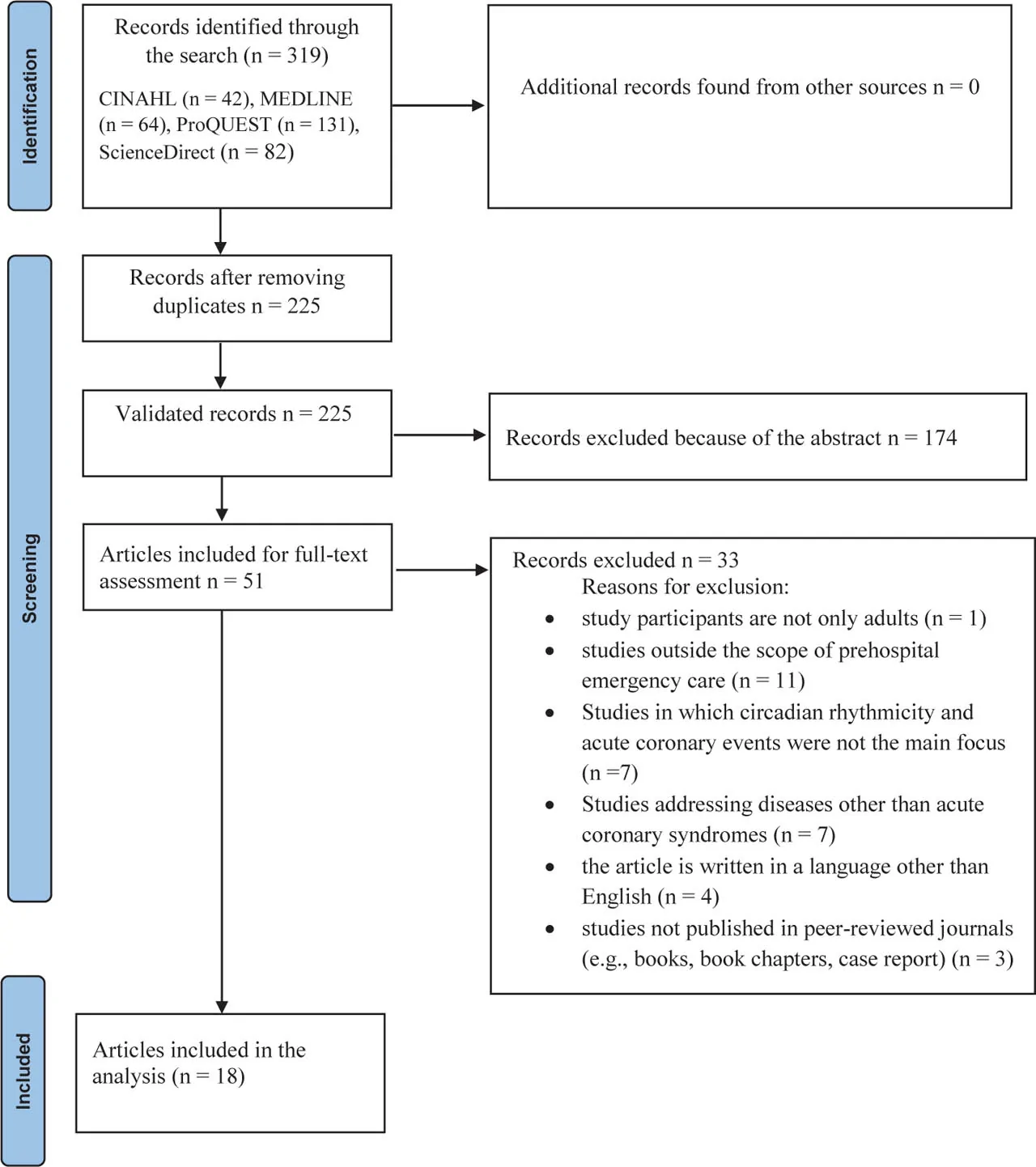
Bootstrap sampling distribution of AUROC (B=1,000 Iteration): group A and B.

**Table 1:** Temporal variation in the onset of OHCA and STEMI in prehospital emergency care

**Authors (year), country**	**Objective**	**Sample**	**Disease**	**Method (time period)**	**Parameters**	**Main findings on circadian rhythms**	**Conclusion**
**Li et al. (2010), China [18]**	To examine circadian, weekly, and age patterns of occurrence of ACS in the prehospital EMS system in Beijing	7032 patients	ACS	Retrospective analysis of electronic prehospital medical records (2005–2007)	Time of onset (24-hours cycle), day of the week, age	A significant variation of circadian distribution of ACS was observed with two peaks (8 a.m. to 10 a.m. and 10 p.m. to 12 a.m.; p < 0.001. This pattern was seen in all age groups; in younger patients (< 65 years), a relatively higher percentage of cases occurred during the morning peak, while in older patients (> 65 years), the occurrence was rather distributed throughout the day. There were no significant variations in the weekly pattern (p = 0.203).	The presentation of ACS has a significant circadian rhythm with morning and night peaks.
**Faramand et al. (2019), USA [17]**	To evaluate diurnal, weekly and seasonal variations of chest pain in patients transported by EMS	2065 patients	Chest pain (ischemic/non-ischemic)	Retrospective analysis of EMS records (2013–2014)	Time of day, day of week, and season of presentation; ischemic vs. non-ischemic causes	A significant circadian rhythm (p <0.001): peak between 8 a.m. and 12 p.m.; minimum at night. Ischemic causes more frequently in the mooring, non-ischemic in the afternoon / evening. Fewer cases during the weekend (p < 0.05). Most cases in winter (p <0.01).	Acute coronary events demonstrate a marked circadian rhythm with a morning peak (8 a.m. to 12 p.m.), when ischemic causes such as myocardial infarction or unstable angina occur most frequently, with more cases in winter and fewer cases at weekends.
**Ni et al. (2019), USA [19]**	To perform a contemporary evaluation of circadian and septadian patterns of the incidence of SCA	1535 patients	SCA	Prospective cohort study; time of first EMS contact (2002–2014)	Time of day (6-hour blocks), day of week, presence of sleep apnea	The traditional morning peak (8 a.m. to 12 p.m.) was not confirmed. A significant early morning nadir (12 a.m. to 6 a.m.; 13.9% of cases; p < 0.001). No Monday peak; a nadir on Sundays (11.3% of cases, p = 0.004). In patients with sleep apnea, the risk of SCA at night was relatively high (statistically significant at p < 0.0001).	The traditional morning peak in the incidence of OHCA was not confirmed; the incidence was lowest at night and on Sundays; patients with sleep apnea had an increased risk also at night.
**Szczerbinski et al. (2020), Poland [20]**	To examine temporal variations in the incidence of OHCA	815 patients	OHCA	Retrospective analysis (2006–2007)	Time of day (1-hour blocks), day of week, season	The peak in incidence between 6 a.m. and 12 p.m. (32.4% of all cases). The lowest number of OHCA cases between 12 a.m. and 6 a.m. (13.0%). The highest number on Saturday (16.1%), the lowest on Friday (10.9%). A peak in winter (December to February; 26%).	OHCA showed a morning circadian peak (6 a.m. and 12 p.m.), the highest incidence on Saturday and the lowest on Friday, and a seasonal peak in winter.
**Szczerbiński et al. (2023), Poland [21]**	To investigate the temporal variability of OHCA occurrence	5058 patients	OHCA	Observational retrospective analysis of the OSCAR-POL registry (2006–2018)	Circadian, weekly, monthly, and seasonal variabilities of OHCA occurrence	The highest OHCA incidence was observed between 8 a.m. and 9 a.m. and the lowest between 1 a.m. and 2 a.m. (p < 0.001). Using 6-hour intervals, the lowest number of OHCA cases was between 12 a.m. and 6 a.m. and the highest between 6 a.m. and 12 p.m. The highest OHCA incidence was in winter, the lowest in summer (p < 0.001). No weekly variability was found. Significant circadian variability was observed for every day of the week, every season and year (p < 0.001).	OHCA showed significant circadian variations with a morning peak at around 8 a.m., the lowest incidence at night, a seasonal peak in winter, and no weekly variability.

ACS – acute coronary syndrome, EMS – emergency medical service, SCA – sudden cardiac arrest, OHCA – out-of-hospital cardiac arrest

**Table 2:** The impact of temporal pattern of OHCA and STEMI on prehospital emergency care – correlations with in-hospital mortality and clinical outcomes

**Authors (year), country**	**Objective**	**Sample**	**Disease**	**Method (time period)**	**Parameters**	**Results**	**Relevance for pre-hospital care**
**Brooks et al. (2010), Canada [23]**	To evaluate temporal variability in the frequency and outcome of OHCA	9667 patients	OHCA of presumed cardiac etiology	Prospective cohort study–EMS records (2005–2007)	OHCA frequency by time blocks (12 a.m. to 6 a.m.; 6 a.m. to 12 p.m.; 12 p.m. to 6 p.m.; 6 p.m. to 12 a.m.); frequency by day of week / month of year; survival to hospital discharge by hour of day / day of week / month of year	OHCA frequency: significant variability by hour of day: compared to the 12 a.m. to 6 a.m. time block, the ORs were 2.02 (95% CI 1.90–2.15) for 6 a.m. to 12 p.m., 2.01 (95% CI 1.89–2.15) for 12 p.m. to 6 p.m., and 1.73 (95%CI 1.62–1.85) for 6 p.m. to 12 a.m. (p < 0.001). Frequency by day of week: statistically significant variation (p = 0.03), with the highest frequency on Saturdays. Frequency by month of year: significant variation (p < 0.001), with the highest frequency in December. Survival to hospital discharge: lowest and when OHCA occurred during the 12 a.m. to 6 a.m. block (7.3%) and highest during the 12 p.m. to 6 p.m. block (9.6%). Survival: highest for OHCA occurring on Mondays (10.0%) and lowest for Wednesday occurrence (6.8%; p = 0.02).	OHCA frequency is highest during the day and in the afternoon, with peaks on Saturdays and in December, while survival is lowest in case of nighttime OHCA, varies by day of week and is highest for OHCA occurring on Monday.
**Holmes et al. (2010), USA [25]**	To assess circadian patterns in STEMI symptom onset and their impact on prehospital delay, timeliness of reperfusion, and mortality	2143 patients	STEMI	Prospective analysis of records (2004–2008)	Time of symptom onset, prehospital delay, door-to-balloon time, in-hospital mortality	Symptoms most commonly occurred from 8 a.m. to 3 p.m. (39%, p < 0.001). Symptom onset between 12 a.m. and 6 a.m. was associated with longer prehospital delays (121 min vs. 70 min for onset 12 p.m. to 6 p.m., p < 0.001) and longer door-to-balloon times (75 vs. 60 min for onset 6 a.m. to 12 p.m., p < 0.001). There was no significant association between time of symptom onset and in-hospital mortality.	Nighttime onset of STEMI is associated with longer prehospital delays and longer time to reperfusion; time of symptom onset has no impact on in-hospital mortality.
**Nakanishi et al. (2011), Japan [31]**	To explore the circadian, weekly, and seasonal variations in the incidence and survival rate of OHCA	2599 patients	OHCA	Prospective multicenter observational study (2004–2008)	Occurrence of OHCA, mortality, circadian, weekly, and seasonal patterns	There were bimodal peaks in the incidence of OHCA: 8 a.m. (6.5%) and 7 p.m. (6.4%); there was a trough at 2 a.m. (1.7%); the differences were statistically significant (p < 0.001). There were no circadian variations in the frequency of witnessed OHCA, bystander CPR, or survival. There was uniformity in the occurrence by day of week, but the rates were highest on Sundays for males and on Mondays for females as well as for patient older than 65 years. There was a significant seasonal variation, with more cases in winter – February was the peak month and July the lowest (71.2% more cases in the coldest months). There were no seasonal variations in mortality; the only exception was better survival for shockable rhythms in the warmest months (56.5% vs. 30.0%, p = 0.04).	OHCA has bimodal circadian peaks in the morning and in the evening, higher incidence in winter; weekly variation is less pronounced; survival is generally independent of the time and season, except for better survival for shockable rhythms in the warmest months.
**Wallace et al. (2013), USA [33]**	To evaluate the effect of time of day on prehospital care and outcomes after OHCA	4789 patients (2008–2012)	OHCA	Retrospective analysis of data from an EMS registry (2008–2012)	Prehospital ROSC, 30-day survival, OHCA incidence	41% of OHCA cases occurred at night. Prehospital ROSC rates were 12.8% and 11.6% for patients with daytime and nighttime OHCA, respectively (p = 0.20). 30-day survival rates were 10.9% and 8.56% for daytime and nighttime OHCA (p = 0.02). After adjustment for demographic and clinical factors, 30-day survival relative risk was 1.10 for daytime cases (95% CI 1.02–1.18, p < 0.05).	Patients who suffered arrests at night were more likely to die than those with daytime arrests. The difference remains even after adjustment for other factors such as age, health status, or time to intervention.
**Karlsson et al. (2014), Denmark [29]**	To investigate diurnal (daytime/nighttime) variations in incidence and outcomes (survival) following OHCA including prior comorbidity and pharmacotherapy	18,929 patients	OHCA of presumed cardiac etiology	National observational study (2001–2010)	Incidence by time periods: 43.9% of cases during daytime (7 a.m. to 3 p.m.), 35.7% during evenings (3 p.m. to 11 p.m.), and 20.6% during nighttime (11 p.m. to 7 a.m.). Clinical and pre-hospital variables: place of arrest (home), severe comorbidities (e.g. COPD), percentage of non-witnessed arrests, proportion of no by-stander CPR, time from recognition of OHCA to rhythm analysis, type of first recorded rhythm. Primary outcome measures: ROSC on arrival at hospital and 1-year survival.	Nighttime patients were more likely to have severe comorbidities (e.g. COPD), arrests at home (87.2% vs. 69.0% day-time / 73.0% evening), non-witnessed arrests (51.2% vs. 48.4% daytime / 43.7% evening), fewer bystander CPR (75.9% vs. 68.4% daytime / 66.1% evening), longer median time from recognition to rhythm analysis (12 min vs. 11 min daytime/evening), and a higher percentage of non-shockable rhythms (80.1% vs. 70.3% day-time / 69.4% evening) – all differences: p < 0.0001. Nighttime patients had lower rates of ROSC on arrival (7.5% vs. 14.8% daytime / 15.1% evening) and 1-year survival (2.8% vs. 7.2% daytime / 7.1% evening), p < 0.0001. Lower 1-year survival of nighttime patients persisted after adjusting for patient- and arrest-related characteristics: OR 0.47 (95% CI 0.37–0.59) and OR 0.51 (95% CI 0.40–0.65) compared to daytime and evening.	Nighttime OHCA patients were more likely to have severe comorbidities, arrests at home, non-witnessed arrests, fewer bystander CPR, and a higher percentage of non-shockable rhythms, leading to considerably lower rates of ROSC and 1-year survival; they were approximately 50% less likely to survive for 1 year than daytime or evening OHCA cases.
**Matsumura et al. (2016), Japan [30]**	To determine whether temporal differences alter survival after OHCA	13,780 patients	OHCA of non-traumatic etiology (cardiac etiology)	Prospective observational study (2012–2013)	Analysis by time of emergency call receipt: daytime (7 a.m. to 3 p.m.), evening (3 p.m. to 11 p.m.), and night (11 p.m. to 7 a.m.); 1-month survival, bystander CPR, call-response interval, defibrillation, ROSC, neurological outcomes	Prehospital ROSC: 10.4% (daytime) vs. 7.4% (evening) vs. 5.5% (night), p < 0.0001; ROSC: 38.2% (daytime) vs. 32.3% (evening) vs. 29.6% (night), p < 0.0001; 24-hour survival: 15.3% (daytime) vs. 10.8% (evening) vs. 9.9% (night), p < 0.0001; 1-month survival: 7.6% (daytime) vs. 5.0% (evening) vs. 4.9% (night), p < 0.0001; 1-month good neurological outcome: 4.7% (daytime) vs. 2.9% (evening) vs. 3.1% (night), p < 0.0001.	ROSC, prehospital ROSC, 24-hour/1-month survival, and good neurological outcome were highest during the day and gradually decreased in the evening and at night. Nighttime OHCA more frequently occurred at home, unwitnessed, characterized by a smaller proportion of shockable rhythms and less bystander CPR.
**Karam et al. (2017), France [28]**	To assess the differences in OHCA characteristics and outcomes according to their time of occurrence (off-hours vs. regular working hours)	9834 patients (2011–2014)	OHCA of non-traumatic etiology	Prospective study (2011–2014)	Analyses comparing arrest characteristics, prehospital care, rhythm, AED use, by-stander CPR, and outcomes (ROSC, hospital admission, survival at discharge)	Off hours were characterized by more frequently present bystanders: 74.4% vs. 72.1% (regular hours), p = 0.01; less frequent bystander CPR: 46.7% vs. 50.6% (regular hours), p = 0.001; less frequently used AED: 1.0% vs. 1.9% (regular hours), p = 0.01; less frequent shockable rhythms: 16.3% vs. 19.1% (regular hours), p <0.0001; less frequent ROSC: 27.5% vs. 31.1%, p <0.0001; lower survival at discharge: 4.7% vs. 6.5%, p <0.0001. Time of occurrence was not associated with worse outcome (OR 0.85; 95% CI 0.69–1.06; p = 0.15).	Off hours were associated with more witnessed OHCA, but less frequent bystander CPR and AED use. Patients were less likely to have shockable rhythms, ROSC and be alive at discharge. Time of occurrence per se was not an independent predictor of poor outcome.
**Jallow, et al. (2018), Sweden [27]**	To describe the temporal variation of OHCA with validated cardiac etiology (myocardial infarction)	3357 patients	OHCA of validated myocardial infarction etiology	Retrospective population study (1989–2009)	Hour of day, day of week, month of year, differences by age and sex, and trends in survival	Total population: significant temporal variation (highest OHCA occurrence between 12 p.m. and 6 p.m., p < 0.0001); difference between days of week (p= 0.0003; highest on Saturdays); difference between months of year (p < 0.0001; highest in January).	Off hours were associated with more witnessed OHCA, but less frequent bystander CPR and AED use. Patients were less likely to have shockable rhythms, ROSC and be alive at discharge. Time of occurrence per se was not an independent predictor of poor outcome.
**Yamashita et al. (2018), Japan [34]**	To investigate diurnal and seasonal variations in survival of OHCA patients, especially elderly individuals, and to identify the effect of mealtimes (breakfast, lunch, dinner) on survival	258,197 patients	OHCA of presumed cardiac etiology	Retrospective analysis of a national OHCA registry (2005–2012)	Number of OHCA cases by hour and mealtime, neurologically favorable 1-month survival, shockable initial rhythm, bystander CPR, and paramedic ALS	Number of OHCAs: elderly (≥ 65 years) – three peaks (breakfast, lunch, dinner), non-elderly (< 65 years) – two peaks (breakfast, dinner). Survival rates lower during nighttime: elderly – 1.6% vs. 2.8%, non-elderly – 7.7% vs. 9.8% (p < 0.01). Dinnertime: significantly lower survival rates in elderly patients only (OR 1.29; 95% CI 1.18–1.41, p < 0.01), non-significant in non-elderly patients (OR 1.02; 95%CI 0.90–1.17). Lower rates of shockable rhythms and bystander CPR at dinnertime for both groups. The season affected decrease in survival rates at dinnertime; the decrease was prominent in winter, not significant during other seasons. Prehospital ROSC and paramedic ALS showed no significant differences.	Dinnertime, especially in elderly patients and during winter, was associated with lower survival rates after OHCA, accompanied by lower rates of shockable rhythms and bystander CPR, while prehospital ROSC and paramedic ALS showed no diurnal variations.
**Schriefl et al. (2019), Austria [32]**	To examine the impact of time of OHCA on clinical outcome in an urban area	1811 patients	OHCA	Prospective cohort study (2013–2015)	ROSC, 30-day survival with favorable neurological outcome, and quality of ALS	ROSC: 30% at daytime vs. 28% at nighttime (p = 0.33); 30-day survival with favorable neurological outcome: 12% at daytime vs. 11% at nighttime (p = 0,51); ROSC: RR 1.1, 95% CI 1.0–1.3, p = 0.19; 30-day survival with favorable neurological outcome: RR 1.2, 95% CI 1.0–1.5, p = 0.10.	There was no significant difference in ROSC and 30-day survival with favorable neurological outcome after OHCA between daytime and nighttime.
**Albackr et al. (2019), Saudi Arabia [22]**	To investigate the association between circadian rhythm and STEMI onset, especially the effect of time of onset on pre-hospital delay and in-hospital mortality	2909 patients	Acute STEMI	Multicenter prospective study (2014–2015)	Time of symptom onset – 24-hour cycle divided into 4 periods: 12 a.m. to 6 a.m., 6 a.m. to 12 p.m., 12 p.m. to 6 p.m., and 6 p.m. to 12 a.m.; prehospital delay and in-hospital mortality assessed	Time of STEMI symptom onset: most frequently during morning and pre-noon hours (6 a.m. to 12 p.m., 30.3%); other times: 12 a.m. to 6 a.m. (23.5%), 12 p.m. to 6 p.m. (23.2%), and 6 p.m. to 12 a.m. (23%); the circadian pattern was confirmed (p < 0.001). Prehospital delay: longest during night and early morning hours: 12 a.m. to 6 a.m. (150 min) and 6 p.m. to 12 a.m. (120 min); shorter during working hours: 6 a.m. to 6 p.m. (90 min); p < 0.001. Reperfusion timeliness: door-to-needle shortest in the morning (p = 0.013), door-to-balloon shortest between 6 a.m. and 12 p.m. (p = 0.007). The association between time of symptom onset and in-hospital mortality was confirmed (p = 0.032).	The onset of STEMI showed a marked circadian pattern with morning and pre-noon peaks. Night and early morning hours were associated with longer prehospital delays and slower reperfusion, while the time of symptom onset had a significant impact on in-hospital mortality.
**Huang et al. (2021), Taiwan [26]**	To investigate the effect of temporal differences on the clinical outcome of OHCA patients	842 patients	OHCA	Retrospective study (2018–2020)	Achievement of ROSC, sustained ROSC, and survival to discharge	Nighttime OHCAs were associated with lower rates of ROSC (OR = 0.489, 95% CI: 0.285–0.840, p = 0.009) and survival to discharge (OR = 0.147, 95% CI: 0.03–0.714, p = 0.017). Subgroup analyses revealed significant temporal differences in males, older adults, patients with longer EMS response times (≥ 5 min), and witnessed OHCA.	OHCAs occurring at night were associated with lower probabilities of achieving survival to discharge and ROSC than daytime arrests. Temporal differences influencing clinical outcomes were more significant in male and older patients, and in OHCAs with longer EMS response time.
**Guo et al. (2024), China [24]**	To assess the circadian rhythm pattern of STEMI onset and the impact of different onset times on clinical outcomes	7805 patients	STEMI	Multicenter prospective study (2017–2019)	Patients divided into 4 groups according to the time of STEMI onset: 12 a.m. to 6 a.m., 6 a.m. to 12 p.m., 12 p.m. to 6 p.m., and 6 p.m. to 12 a.m.; analysis by day of week, quarter of year, patient subgroup, in-hospital mortality, clinical outcome, and 1-year MACCE	STEMI onset showed a bimodal circadian distribution: primary peak at 8.38 a.m. and secondary peak at 12.55 p.m. (P < 0.001). The two-peak pattern was consistent on all days of week and in most quarters of year (Q1–Q3). There was no second (noon) peak in the last quarter (Oct–Dec). No difference between males and females were observed. The time of STEMI onset affected neither in-hospital mortality nor 1-year MACCE.	STEMI onset exhibited a steady bimodal circadian with peaks in the morning and at noon; the latter was missing in the last quarter of year. This temporal pattern is not influenced by sex and has no impact on in-hospital mortality or adverse cardiovascular events during the first year.

OHCA – out-of-hospital cardiac arrest, EMS – emergency medical service, OR – odds ratio, CI – confidence interval, STEMI – ST-segment elevation myocardial infarction, CPR – cardiopulmonary resuscitation, ROSC –return of spontaneous circulation, COPD – chronic obstructive pulmonary disease, AED – automated external defibrillator, ALS – advanced life support, RR – relative risk, MACCE – major adverse cardiovascular and cerebrovascular events

For the purposes of this review, the articles were divided according to their focus in order to distinguish clearly between studies evaluating only circadian or temporal variability in event occurrence and those that also considered clinical outcomes. Five studies (Table 1) focused exclusively on temporal variability [17,18,19,20,21]. The other 13 studies (Table 2) monitored, in addition to incidence, parameters such as survival, return of spontaneous circulation, mortality, or reperfusion [22,23,24,25,26,27,28,29,30,31,32,33,34].

## Results

**a) Study characteristics**

A total of 18 studies published between 2010 and 2024 were included in the review. Collectively, they enrolled more than 250,000 patients, and the cohort sizes varied considerably – from small cohorts with a limited number of patients [20] to large national registries containing thousands of records [29,34]. Most studies focused on OHCA in various geographical locations, including Canada, the USA, Japan, Denmark, and Poland. A smaller group of studies analyzed myocardial infarction, primarily in China, the USA, Saudi Arabia, and Canada. One study examined broader symptoms, namely chest pain regardless of definitive diagnosis [17]. In terms of design, retrospective observational analyses based on emergency medical service registries or hospital databases predominated. A smaller proportion were prospective multicenter studies [24,28,30]. A specific group comprised studies utilizing national registries, which made it possible to conduct robust population analysis of temporal variability [29,31,19].

b) Temporal variation in the onset of OHCA and STEMI in prehospital emergency care

Of the five studies analyzed, three [17,20,21] consistently confirmed the existence of significant circadian variability, with most cases occurring in the morning (especially between 6 a.m. and 12 p.m.) and a minimum occurring at night. One study [18] confirmed the morning peak but was also the only study to identify a nighttime peak (10 p.m. to 12 a.m.). These findings indicate an increased incidence of acute coronary events in the morning, which aligns with previously described circadian variations in cardiovascular physiology, including changes in autonomic tone, blood pressure, heart rate, and haemostatic activity upon awakening. In contrast, one study [19] failed to confirm the classic morning peak, as cases were distributed rather evenly throughout the day. At the same time, the authors emphasized that, in specific subgroups such as patients with sleep apnea, the typical nighttime decline was not observed. This suggests that the presence of comorbidities can significantly modify the influence of circadian rhythms on the time of onset of acute events.

Some studies also assessed seasonal variability, with three studies [17,20,21] showing higher incidence rates in winter and lower rates in summer. Regarding weekly variability, the results were inconclusive. Some studies reported a lower incidence on weekends[17,20], while a long-term analysis [21] found no differences across the week.

Overall, the majority of studies demonstrate a clear circadian pattern in the incidence of acute coronary events, characterized by a morning peak and nocturnal nadir, with seasonal modulation suggesting an additional environmental influence.

**c) The impact of temporal pattern of OHCA and STEMI on prehospital emergency care – correlations with in-hospital mortality and clinical outcomes**

An analysis of the identified studies confirmed the significant temporal variability in the incidence of OHCA. A morning peak (mostly between 6 a.m. and 12 p.m., often with a maximum around 8 a.m.) was found in most studies [23,26,29,30,31,34]. Another, secondary afternoon or evening, peak (bimodal distribution) was mentioned by several authors [23,27,31,34], while others showed a single morning peak [29,30].

In STEMI, the onset of symptoms was most common in the morning [22,24,25]. One study [24] revealed a bimodal distribution of STEMI, featuring primary and secondary peaks at 8:38 a.m. and 12:55 p.m., respectively. This pattern remained consistent across most days and seasons. The incidence of STEMI was lowest at night (12 a.m. to 6 a.m.). Nighttime and early morning cases had longer time intervals to intervention, significantly affecting prehospital delays and reperfusion times [22,25]. In-hospital mortality for STE-MI was lowest among patients whose symptoms began between 4 a.m. and 10 a.m. Conversely, the onset of symptoms at other times correlated with a higher risk of death during hospitalization [22].

Time of symptom onset influenced outcomes in both conditions; however, most studies indicated that time itself is not an independent predictor of prognosis. Patients with nighttime OHCA were less likely to experience return of spontaneous circulation and had worse one-month and one-year survival rates and poorer neurological outcomes [26,29,30]. Instead, outcomes were mainly determined by factors such as witnessed collapse, bystander CPR, shockable rhythm [26,28,29,30], and access to reperfusion therapy [22,25]. A French study [28] emphasized that lower survival rates during off-hours were primarily due to the limited availability of bystander resuscitation and the use of automated external defibrillators rather than the time of onset. In contrast, an Austrian study[32] conducted in an urban setting failed to find differences in survival depending on off-hours.

Seasonal variability was particularly evident in OHCA, with a higher incidence in the winter months [31,34]. Weekly variations were less pronounced, and mortality or survival did not differ statistically significantly by day [23,27].

Overall, the evidence indicates a consistent temporal pattern in both OHCA and STEMI, characterized by a morning peak and nocturnal nadir. However, prognosis is predominantly determined by system-related and clinical factors rather than timing alone.

## Discussion

Our review indicates that temporal variation in acute coronary events and out-of-hospital cardiac arrest is primarily explained by underlying physiological, behavioral, and environmental mechanisms rather than timing itself as an independent determinant.

Circadian variation in cardiovascular events is well established and reflects dynamic changes in autonomic nervous system activity, hemostatic balance, endothelial function, and inflammatory pathways, which may transiently increase vulnerability to acute coronary events [35,36,37,38]. These mechanisms act in a time-dependent manner, resulting in predictable periods of increased cardiovascular instability.

Behavioral and external factors may further contribute to these patterns, as physical exertion, emotional stress, and daily activity levels can act as triggers in susceptible individuals. These influences likely interact with underlying physiological vulnerability and help explain the clustering of events during specific periods of the day without implying a direct causal role of time itself. Worse clinical outcomes observed in nighttime events appear to be predominantly driven by system-level factors rather than biological timing. Reduced likelihood of witnessed collapse, lower rates of bystander cardiopulmonary resuscitation, delayed defibrillation, and decreased availability of immediate medical care during off-hours all contribute to delays in recognition and treatment, ultimately affecting survival and neurological outcomes [30,39,40,41].

Seasonal variation in cardiovascular events is also evident and likely reflects a complex interplay of environmental and physiological influences. Factors such as low ambient temperature, air pollution, and meteorological variability may increase cardiovascular stress and contribute to triggering acute events during colder periods [42,43,44,45,46].

Importantly, these findings indicate that temporal patterns of cardiovascular events are not limited to pre-hospital settings but are also relevant in high-risk hospitalized populations. In critically ill patients, circadian variation has been demonstrated in arrhythmias and cardiac arrest occurrence [47,48] while disrupted circadian regulation, including abnormal blood pressure patterns, has been associated with increased mortality [49]. Similarly, in postoperative patients, cardiovascular complications show a distinct temporal distribution, with the highest risk occurring in the early post-operative period [50], and specific complications such as postoperative atrial fibrillation demonstrating time-dependent onset patterns [51]. These findings confirm that temporal variability is clinically relevant across high-risk in-hospital populations. From a clinical perspective, these observations support the need for targeted in-hospital monitoring and increased awareness during periods of higher vulnerability, which may facilitate earlier detection and timely intervention, as well as optimization of staffing and surveillance strategies. However, further research is needed to determine whether temporally tailored monitoring approaches translate into improved clinical outcomes.

## KEY FINDINGS

- **Circadian pattern:** The incidence of OHCA and STEMI shows a consistent circadian distribution, with a predominant morning peak (6 a.m. to 12 p.m.) and a nocturnal nadir; OHCA may additionally exhibit a secondary afternoon/evening peak.
- **Outcomes and system factors:** Nighttime events are associated with worse short- and long-term outcomes; however, this appears to be mainly explained by reduced bystander intervention, lower rates of CPR/defibrillation, and longer delays to treatment rather than the timing of onset itself.
- **Seasonal and weekly variation:** A higher incidence in winter suggests an environmental contribution to event triggering, whereas weekly variation appears inconsistent and less clinically relevant across studies.

## Study limitations

The main limitations of the present study are heterogeneity of the available data and the ambiguous definitions of acute coronary events. Varied measurement methods may affect the comparability of results. Additionally, the conclusions cannot be generalized to other populations as the included studies differed in age distribution, comorbidities, and the geographical and organizational context of prehospital care.

## Conclusion

Temporal patterns are consistently observed in OHCA and STEMI, particularly a morning predominance and lower incidence during nighttime, with additional seasonal variation suggesting environmental influence. Clinical outcomes are influenced more by system-level factors than by the timing of events itself. These findings underline the importance of temporal awareness in emergency care, while emphasizing that prognosis is primarily determined by the speed and quality of the response system.
